# Distribution of the Quill Mite *Bubophilus asiobius* Parasitizing Western Palaearctic Owls of the Genus *Asio*

**DOI:** 10.1155/japr/5550682

**Published:** 2025-11-17

**Authors:** Zbigniew Kwieciński, Adam Linkowski, Jan Hušek, Maciej Skoracki

**Affiliations:** ^1^Department of Avian Biology and Ecology, Faculty of Biology, Adam Mickiewicz University, Poznań, Poland; ^2^Department of Animal Morphology, Faculty of Biology, Adam Mickiewicz University, Poznan, Poland; ^3^Department of Zoology, National Museum of the Czech Republic, Prague, Czech Republic

**Keywords:** Acari, ectoparasites, host-parasite relationship, Long-eared Owl, Marsh Owl, quill mites, Short-eared Owl, Syringophilidae

## Abstract

**Background:**

Birds and their parasites have coevolved over millions of years, forming intricate relationships that shape biodiversity. Until recently, little was known about the mites parasitizing owl feathers. Quill mites of the family Syringophilidae (Acariformes: Prostigmata) are highly specialised ectoparasites that reside within feather quills. Despite their widespread occurrence, their diversity, distribution and host associations remain poorly understood. This study investigated the distribution and ecological interactions of the quill mite *Bubophilus asiobius* in the feather quills of Western Palaearctic owls of the genus *Asio*: the Long-eared Owl (*Asio otus*), Marsh Owl (*Asio capensis*) and Short-eared Owl (*Asio flammeus*).

**Methods:**

A total of 196 owl specimens were examined. Feathers were categorised as follows: primaries (P), secondaries (S), rectrices (T), primary greater upper-wing coverts (PGUppC), secondary greater upper-wing coverts (SGUppC), alula feathers (Af) and scapular greater coverts.

**Results:**

Prevalence was relatively low across hosts: 9.3% (CI = 4.9%–16.5%; *N* = 108) in Long-eared Owl, 9.3% (CI = 4.5%–18.5%; *N* = 75) in Short-eared Owl and 0% in Marsh Owl (*N* = 13). In total, 27,440 flight feathers and coverts were inspected. *Bubophilus asiobius* was detected in primaries, secondaries, rectrices, PGUppC, SGUppC, Af and scapular greater coverts of Long-eared and Short-eared Owls. No mites were found in Marsh Owls.

**Conclusion:**

Our findings indicate that *Bubophilus asiobius* exploits a broader spectrum of feather types in its hosts. We documented infestations in primaries, small inner secondaries, rectrices, Af, as well as in the primary and secondary greater coverts and scapular greater coverts of both the Long-eared Owl and the Short-eared Owl. The frequent occurrence of mites in primaries, secondaries and rectrices suggests that these feather types provide particularly favourable conditions for colonisation. The primary factor determining mite spread appears to be the availability of newly growing feathers. During feather growth, female mites colonise available quills and penetrate the walls irrespective of their thickness, enabling unrestricted foraging. Thus, quill size and wall thickness influence the number of mites that can establish within a feather but do not limit feather suitability for infestation. An alternative explanation is that the association between *Bubophilus asiobius* and owls of the genus *Asio* represents a relatively recent and ecologically unstable host–parasite relationship.

## 1. Introduction

**Figure 1:**
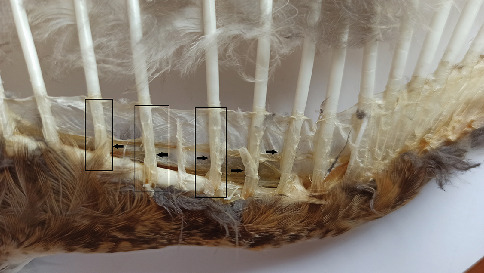
The space (marked by an arrow) where the quill mite *Bubophilus asiobius* colonies live.

Quill mites of the family Syringophilidae (Acariformes: Prostigmata: Cheyletoidea) are highly specialised avian parasites that occur exclusively in feather quills [1, 2]. These mites exhibit a high degree of host specificity, with most species being either monoxenous or oligoxenous [3, 4]. They also show distinct preferences for the specific habitats they colonise [5, 6]. Syringophilid mites feed on soft tissue fluids of their avian hosts [4, 6] (see Figure 1). Consequently, successful reproduction and colony formation are restricted to particular feather types [5, 7, 8].

The genus *Bubophilus* was established for single species *Bubophilus ascalaphus*, described from the Great Horned Owl *Bubo virginianus* (Strigiformes: Strigidae) in the United States [9, 10].

To data, approximately 443 species of Syringophilidae, grouped into 62 genera, have been described, and the range of their avian hosts comprises 24 orders from all zoogeographical regions except Antarctica [11]. Despite this diversity, knowledge of syringophilid mites associated with owls remains limited [11, 12]. Thus far, only eight species have been reported from 16 owl species, including representatives of *Bubophilus* (five species), *Neobubophilus* (2) and *Megasyringophilus* (1). These parasites have been found on hosts belonging to both Strigidae—*Bubo bubo* (Eagle Owl), *Bubo africanus* (Spotted Eagle-Owl), *B. virginianus* (Great Horned Owl) [9]; *Asio otus* (Long-eared Owl) [10]; *Asio flammeus* (Short-eared Owl) [present study]; *Strix aluco* (Tawny Owl), *Strix woodfordii* (African Wood Owl) [13, 14]; *Strix uralensis* (Ural Owl), *Strix nebulosa* (Great Grey Owl) [15]; *Aegolius funereus* (Tengmalm's Owl) [16]; *Megascops choliba* (Tropical Screech-Owl); *Athene noctua* (Little Owl), *Athene brama* (Spotted Owlet), *Athene cunicularia* (Burrowing Owl) [12]—and Tytonidae—*Tyto alba* (Barn Owl) [9] and *T. a. affinis* (African Barn Owl) [12].

The evolutionary history of modern *Strigidae* genera remains unclear. The earliest known representative, *Asio brevipes*, originates from the Upper Pliocene of Idaho [17, 18]. Fossil remains of Long-eared Owl have been recovered from numerous Pleistocene sites across Europe and North America [17–19].

The Long-eared Owl *A. otus* belongs to the Holarctic faunal type and has a wide circumpolar distribution across boreal, temperate, Mediterranean and steppe climatic zones [18, 19]. The Old World birds are generally paler, more finely barred on the underparts and more extensively streaked below. Although largely sedentary, northern populations exhibit southward and westward migratory movements in winter [19, 20].

The Short-eared Owl *A. flammeus* is one of the world's most widely distributed owls. An open-country, ground-nesting species, it inhabits marshes, grasslands and tundra across much of North America and Eurasia. Data on populations outside the nominate race *A. f. flammeus* remain scarce [20, 21], with most other subspecies restricted to islands. The species belongs to the Holarctic faunal type, with a circumpolar distribution and a discontinuous range in South America [18, 19]. The populations on isolated island groups, such as Hawaii, the Galápagos and Caroline Islands, likely originated through the long-distance dispersal movements characteristic of this species [20, 21].

The Marshall Owl *Asio capensis* represents the Ethiopian fauna and is sometimes referred to as the Algerian Marsh Owl. Its breeding range extends from North Africa to the Cape, where it is locally common [20, 22]. This species occupies a variety of open habitats, including coastal grasslands, marshlands, montane grasslands up to 3000 m, rice fields and lightly wooded savanna, while avoiding dense forest. Its distribution is influenced by annual rainfall fluctuations, which periodically shift its range into drier grassland and bushveld. The species occurs from northwestern Morocco and scattered localities in West Africa (Senegal to Chad and Cameroon), through South Sudan and the Ethiopian Highlands, to southern Congo, southern Democratic Republic of the Congo, Namibia, northern Botswana and much of South Africa, extending as far south as the Cape [18, 19]. The Marsh Owl is a partially migratory species, although its nonbreeding movements remain poorly understood. Some populations vacate breeding areas during the wet season, and the species is a confirmed nonbreeding visitor to coastal Gambia. Vagrants have been recorded in Spain, Portugal and Canary Island [20, 22].

This study presents new data on the distribution of the parasitic quill mite *Bubophilus asiobius* (Acariformes: Syringophilidae) in the plumage of the Western Palaearctic owls of the genus *Asio*. Furthermore, we discuss the possible mechanisms of mite distribution and dispersal, as well as their occurrence in relation to plumage structure and habitat preferences.

## 2. Materials and Methods

Mite material used in this present study was collected from dry bird skins and wings housed in the Natural History Collections (AMU-DABE) of the Faculty of Biology, Adam Mickiewicz University in Poznań, Poland. Specimens consisted of either whole dry wings or a full complement of flight feathers and greater coverts. Feathers from 108 Long-eared Owl, 75 Short-eared Owl and 13 Marsh Owl were analysed in the study. The following abbreviations are used for feather types: primaries (P), secondaries (S), rectrices (R), primary greater upper-wing coverts (PGUppC), secondary greater upper-wing coverts (SGUppC), alula feathers (Af) and scapular greater coverts [23–25]. Feather samples were subsequently examined for the presence of mites in the family Syringophilidae.

The feathers were subsequently examined for the presence of quill mites of the family Syringophilidae, species *B. asiobius*. Infested quills were placed in Nesbitt's solution at 50°C for 1 h to soften the mites contained within. Each quill was then carefully opened along its length using fine-tipped forceps. The mites were rinsed in 70% ethanol and mounted on permanent microscope slides using Faure's medium [15, 26]. Slide-mounted mites were examined with a ZEISS Axioscope light microscope (Carl Zeiss AG, Oberkochen, Germany) equipped with differential interference contrast (DIC) optics and a camera lucida. Prevalence values and their exact 95% confidence intervals (Sterne's method, confidence level = 95%) were calculated using Quantitative Parasitology on the Web [27, 28]. Mites were identified to species level using the identification key [12] and the original description provided by Skoracki and Bochkov [10].

All collected mite specimens have been deposited in the Department of Animal Morphology, Adam Mickiewicz University, Poznań, Poland (AMU). Feather samples are currently housed in the Natural History Collections and the Department of Avian Biology and Ecology, Faculty of Biology, Adam Mickiewicz University, Poznań, Poland (AMU-DABE).

## 3. Results

In total, 27,440 feathers from 196 individuals of the three species, Long-eared Owl, Short-eared Owl and Marsh Owl were examined. In all infested samples, the quill mite *B. asiobius* was identified [10].

### 3.1. Prevalence and Feather Infestation of the Long-Eared Owl

In total, 15,120 feathers from 108 specimens (host no. AMU-DABE AO 1-108; 51 males—22 juveniles and 29 adults and 57 females—29 juveniles and 28 adults) were analysed (Table 1). These owls originated from Poland, Germany, Slovenia, Slovakia, Spain, Turkey, Ukraine, France, Iceland and Greece. Of these, only 10 individuals were infested with quill mites, yielding a prevalence index (IP) of 9.3% with a 95% confidence interval (Sterne's method) of 4.9%–16.5% (Table 2).

Among the examined material, consisting of 51 juveniles (1st calendar year [CY]) and 57 adults (2nd CY or older), only four juveniles (IP = 7.8%, CI = 2.7%–18.1%) and six adults (IP = 10.5%, CI = 4.7%–21.7%) were infested with quill mites. The infested owls included the following: two juvenile males from Poland, one juvenile female from Germany, one juvenile female from Poland, one adult male from Poland, one adult male from Iceland, three adult females from Poland and one adult female from Ukraine.

**Figure 2:**
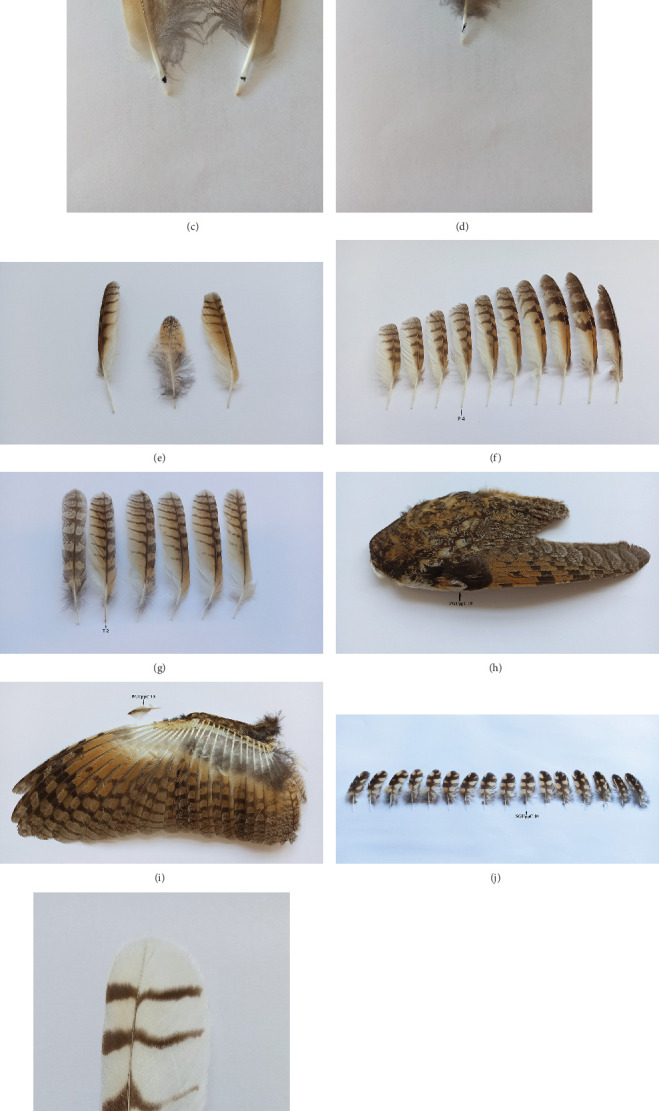
Feathers (marked by an arrow and black dot) infested with quill mites, *Bubophilus asiobius* sp. n. (a) The Long-eared Owl (ad. female; host no. AO 24 from Poland), primaries—P2–P6 (right wing) and P5–P7 (left wing). (b) The Long-eared Owl (ad. female; host no. AO 24 from Poland), secondary—S14. (c) The Long-eared Owl (ad. female; host no. AO 24 from Poland), alula feathers: Af no. 1 – left and 1 – right wings. (d) The Long-eared Owl (ad. female; host no. AO 24 from Poland), secondary greater upper-wing covers: SGUppC no. 3. (e) The Long-eared Owl (ad. female; host no. AO 57 from Poland), from the left: primaries—P2, scapular greater coverts: no. 1, rectrices—T6. (f) The Long-eared Owl (ad. male; host no. AO 75 from Iceland), primaries—P4. (g) The Long-eared Owl (ad. female; host no. AO 99 from Crimea), rectrices—T2. (h, i) The Long-eared Owl (juv. male; host no. AO 103 from Poland), primary greater upper-wing coverts—PGUppC no. 10; H—wing with covers, I—wing without covers. (j) The Short-eared Owl (ad. female; host no. AF 3 from Poland), secondary greater upper-wing covers: SGUppC no. 10. (k) The Short-eared Owl (ad. female; host no. AF 7 from Slovenia), rectrices—T4. (l) The Short-eared Owl (juv. male; host no. AF 46 from Germany), primaries—P1 and P8.

Quill mites *B. asiobius* were found in 26 feathers. The mite material examined, collected from the feather quills of the Long-eared Owl, is presented in Table 3 (Figures 2a, 2b, 2c, 2d, 2e, 2f, 2g, 2h and 2i).

### 3.2. Prevalence and Feather Infestation of the Short-Eared Owl

A total of 75 specimens (host no. AMU-DABE AF 1-75; 40 males—23 juveniles and 17 adults and 35 females—12 juveniles and 23 adults) originating from Poland, Slovenia, Denmark, Finland, Greece, Sweden and Iceland were examined. In total, 10,500 feathers were analysed (Table 1). Of these, seven host individuals were infested with quill mites, yielding a prevalence (IP) of 9.3% (CI = 4.5%–18.5%; Table 2).

The material included 35 juveniles (1st CY) and 40 adults (2nd CY or older). Among them, two juveniles (IP = 6.1%, CI = 1.1%–19.4%) and five adults (IP = 15%, CI = 6.7%–29.8%) were infested. The infested individuals comprised: one juvenile male from Poland, one juvenile male from Germany, one adult female from Poland, one adult female from Slovenia, one adult female from Iceland, one adult male from Poland and one adult male from Greece.

Quill mites were found in nine individual feathers. The mite material examined, collected from the feather quills of the Short-eared Owl, is presented in Table 3 (Figures 2j, 2k and 2l). This species is a new host for *B. asiobius*.

### 3.3. Prevalence and Feather Infestation of the Marsh Owl

In total, 1820 feathers were analysed (Table 1) from 13 specimens (host no. AMU-DABE AC 1-13; seven males and six females) originating from Morocco. In the material consisting of eight juveniles (in the 1st CY) and five adults (in the 2nd CY or older), all host specimens were uninfested by quill mites IP = 0% (Table 2).

## 4. Discussion

*B. asiobius* was originally described from the Long-eared Owl, *A. otus*, in Poland [10]. The new record presented in this paper—its occurrence in the Short-eared Owl *A. flammeus*—demonstrates that this quill mite species is not monoxenous and restricted to a single host, but rather oligoxenous, parasitising closely related species within the genus *Asio*. Unfortunately, we were unable to confirm its presence in a third potential host of the genus, the Marsh Owl *A. capensis*.

Among avian hosts, the index of prevalence (IP) shows considerable variation, ranging from 1.4% to 100% [29, 30]. Such differences may reflect host behaviour [31–33], habitat characteristics [4, 7, 32] or the strength and specificity of host immune responses to infestation [29, 32, 34]. In the present study, the prevalence of quill mite infestation was relatively low, between 7.8% and 9.3%. Nevertheless, these values are consistent with prevalence levels commonly reported for non-social, independently breeding birds [31, 34, 35]. In such hosts, opportunities for parasite transmission are limited, as individuals neither form colonies nor engage in close social interactions that would facilitate mite dispersal. This suggests that mites relying on transmission routes other than direct transfer through nestlings or copulation have few opportunities to colonise new hosts [29, 34].

By contrast, in gregarious hosts, the prevalence of infestation is usually much higher, with the highest values recorded in social and domesticated birds [34, 35], where horizontal transmission is thought to play a major role in the spread of quill mites [30, 32, 36]. Research on syringophilid mites parasitising owls remains scarce [11, 12], and data on infestation levels in owl populations are limited. Current findings indicate that prevalence in owls is generally low, usually between 2% and 12% [15, 16]. For instance, *Bubophilus aegolius* was recorded in the Tengmalm's Owl *A. funereus* with an IP of 7.3% (*N* = 55) [15]. Similarly, *Bubophilus aluconis* was reported with an IP of 12% in the Ural Owl *S. uralensis* (*N* = 79), 2.6% in the Tawny Owl *S. aluco* (*N* = 77) and 3.6% in the Great Grey Owl *S. nebulosa* (*N* = 55) [16].

The consistently low prevalence of *B. aegolius*, *B. aluconis* and *B. asiobius* suggests that these mites exploit only a small fraction of the available host populations [15, 16]. This pattern may be explained by the generally solitary behaviour of their owl hosts, which likely restricts opportunities for mite transmission [19, 20].

The situation may differ between the Long-eared Owl and the Short-eared Owl, as both species display varying degrees of social behaviour. The Long-eared Owl is primarily sedentary, although the northernmost breeding populations typically migrate south to temperate regions during winter [17, 21]. Resident birds occupy their territories year-round and are found in forests, woodlands and scrub, though usually on the edges of larger wooded areas [20, 21]. Most pairs breed in small woodland patches among open meadows and fields [17, 20], typically nesting in abandoned stick nests of other bird species [17, 18]. Clutch size averages four to five eggs (range 1–10), depending on prey availability and the female's condition [19, 20]. During the breeding season, pairs may engage in mutual preening (allopreening), particularly around the head, immediately before and after copulation [37]. Allopreening is also observed during brood-rearing and probably continues throughout the nesting period [37, 38]. Communal roosts form from June to October and disperse by February or March, usually consisting of 2–20 birds, although gatherings of up to 100 individuals have been reported [19].

The Short-eared Owl, the second host species, is a widely distributed open-country owl that inhabits marshes, grasslands and tundra across much of North America and Eurasia. Unlike most owls, it constructs its own nest on the ground, lined with grasses and feathers [20, 21]. Clutch size ranges from two to 13 eggs. It is a partial migrant, with northern populations moving southward in search of food. Preening behaviour involves spreading oil from the uropygial gland and realigning feathers with the bill. Short-eared Owls may roost in trees either alone, communally or even interspecifically, often alongside Long-eared Owls [18, 19]. During the non-breeding season, they may form large ground roosts, with up to 200 individuals recorded together [20, 21].

By contrast, little is known about the ecology and biology of the Marsh Owl. Ecologically, it resembles the Short-eared Owl, occupying open grassland or marsh habitats, but is more nocturnal. It nests on the ground in tunnels forced through vegetation or in hollows beneath large tufts [18, 19], lining the nest with dry grass and hay. Breeding in Morocco typically begins in April, with clutches of two to four (occasionally up to six) eggs [20, 21].

The high reproductive output of these owls, coupled with frequent interactions among adults both during and outside the breeding season, likely creates favourable conditions for mite transmission and establishment. In the system comprising *B. asiobius*, the Long-eared Owl, and the Short-eared Owl, horizontal transfer—if it occurs—may play an important role in mite dispersal. For the Marsh Owl, however, no such information is available. Within the sampled Long-eared Owl population, quill mites were detected in only four juveniles (first CY, 1CY) and six adults (second CY, 2CY or older). In the Short-eared Owl, two juveniles and five adults were infested. No infestation was found in either juvenile or adult Marsh Owls.

This absence may be explained by differences in host moulting dynamics [4–6]. Adult syringophilid females typically abandon fully developed quills and migrate to colonise newly forming feathers [39–41]. In the Long-eared and Short-eared Owls, moult occurs from late June–July to November or December [17, 19, 21], whereas in the Marsh Owl, it extends from late January or April to May or July [28, 38]. For successful transmission, mite dispersal must coincide with host feather replacement, as the detachment of an old feather does not necessarily align with the immediate growth of a new one [6, 8, 23, 39–41].

The lack of mites in the examined Marsh Owls does not exclude the possibility of vertical transmission between adults and offspring. More likely, it reflects sampling limitations or the absence of documented infestations to date.

### 4.1. Habitat Preference

The processes by which dispersing mites select and colonise suitable feathers remain poorly understood [30, 32, 42, 43]. Feather suitability for a given mite species appears to depend largely on two structural features: quill volume and wall thickness [4, 6, 30, 44]. At present, feather moult is regarded as the principal mechanism facilitating intra-host transmission of quill mites [4, 8, 29, 30]. In the wild, owls of the genus *Asio* exhibit a relatively consistent moulting sequence [45, 46].

In owls, moult follows a structured pattern. Primary feathers are replaced first, beginning with P1 and progressing sequentially to P10. Secondary feathers are replaced from three centres (S1, S5 and S11 or S12), though their replacement is variable: while most are shed in a given season, some (S3, S4, S6–S8) may remain unmoulted. Rectrices are typically lost later in the season within a short period, followed by head and belly feathers [23, 38, 39, 45, 46]. In both nestlings and adults, inner secondaries (S13–S16), primary and secondary coverts, under-tail coverts and scapulars are among the first feathers to complete growth [23, 39].

Previous studies indicate that *B. aluconis* and *B. aegolius* infest only a small proportion of their potential host populations, likely due to the solitary behaviour of owls [15, 16]. Moreover, these species appear to occupy a relatively narrow set of feather types (inner secondaries and wing coverts). By contrast, our results show that *B. asiobius* utilises a broader range of feathers in its hosts. We recorded infestations in primaries, small inner secondaries, rectrices, alula feathers, primary and secondary greater coverts and scapular coverts of the Long-eared Owl and the Short-eared Owl. The frequent occurrence in primaries, secondaries and rectrices suggests that these feather types offer particularly favourable conditions for colonisation [30, 40, 41, 43, 44].

The spread of *B. asiobius*, however, seems primarily determined by the availability of newly growing feathers [15, 16, 30, 36, 42]. Feathers remain flexible for several days after completing growth and require additional time to lose residual moisture and fully harden [23, 47]. During this interval, female mites can penetrate the quill wall and forage effectively [4, 5, 43]. Some studies even suggest that moulting does not necessarily reduce mite loads; in fact, mite abundance may increase during this period [48, 49]. Quill mites may detect feathers about to be shed and migrate to adjacent ones, thereby avoiding loss with discarded feathers [47, 50–52].

In addition, self-preening (auto grooming) and mutual preening (allopreening) may facilitate the horizontal transfer of mites, along with other ectoparasites, bacteria and viruses [47, 53–57]. Such behaviour has been documented in both Long-eared and Short-eared Owls [17, 19, 21, 37]. Our findings of mites in very small feathers—including alula feathers, greater coverts and scapular greater coverts (see Figure 1)—support the view that preening contributes to their redistribution across the body, reaching feather types usually less accessible. We therefore propose that preening constitutes an additional mechanism of feather infestation in *Asio* owls.

## 5. Conclusion

The relatively low prevalence of *B. asiobius* is likely shaped by multiple interacting factors, including the timing and pattern of host moult, the structural properties of feathers, and the behavioural and reproductive traits of Long-eared and Short-eared Owls. An alternative explanation, however, should also be considered. The association between *B. asiobius* and members of the genus *Asio* may represent an evolutionarily recent and ecologically unstable host–parasite relationship. In this context, *B. asiobius* could be viewed as a species that has only recently expanded its host range, yet achieves limited success in survival, reproduction and dispersal within these novel hosts. To evaluate this hypothesis, further investigations of quill mite assemblages across the remaining nine *Asio* species are needed [57–59].

## Figures and Tables

**Table 1:** Number and type of feathers examined.

**Type of the feathers (** **N** **)**	***Asio otus* (** **N** = 108**)**	***Asio flammeus* (** **N** = 75**)**	***Asio capensis* (** **N** = 13**)**
Primaries *N* = 10	2160	1500	260
Secondaries *N* = 16	3456	2400	416
Rectrices *N* = 12	1296	900	156
Primary greater upperwing covert (PGUppC), *N* = 10	2160	1500	260
Secondary greater upperwing covert (SGUppC), *N* = 14	3 024	2100	364
Alula feathers (Af), *N* = 4	864	600	104
Scapular greater covert, *N* = 10	2160	1500	260

**Table 2:** Host species of the genus *Asio* infested with quill mite *Bubophilus asiobius* with the prevalence, types of infested feathers and distribution.

**Host species**	**No of examined/infested**	**Prevalence and confidence interval**	**Type of infested quill feathers**	**Locality**
*Asio otus*	108/10	9.3%; 4.9–16.5	Primaries, inner secondaries, rectrices, alula feathers, primary greater upper-wing coverts (PGUppC), secondary greater upper-wing coverts (SGUppC), covers from the scapulars	Poland, Iceland, Germany, Ukraine
*Asio flammeus*	75/7	9.3%; 4.5–18.5	Primaries, secondaries, rectrices, secondary greater upper-wing coverts (SGUppC)	Poland, Slovenia, Iceland, Germany, Greece
*Asio capensis*	13/0	0%	—	Morocco

**Table 3:** Examined material of quill mites *Bubophilus asiobius* collected from the feather quills of the Long-eared Owl and Short-eared Owl.

**Host number (age, sex)**	**Number of male quill mites**	**Number of female quill mites**	**Habitat**	**Location-region, town, country**	**Date of specimen collection**
AO 24 Adult, Female	34	247	P2-P4, P5(l,r), P6(l,r), P7, S14, SGUppC3, Af1(l,r)	Poland: Kampinos	12.10.2002
AO 57 Adult, Female	3	20	P2, T6, coverts from scapulars 1	Poland: Lądek	12.04.2003
AO 68 Adult, Male	11	104	P2, P4, P5	Poland: Lublin	04.02.2002
AO 69 Juv. Female	4	33	P5	Poland: Lublin	13.05.2003
AO 75 Adult, Male	1	15	P4	Iceland: Ostisland	07.04.2003
AO 77 Juv. Female	1	10	T1	Germany: Osten	23.09.2003
AO 97 Juv. Male	0	3	P1	Poland: Michów	10.02.2006
AO 99, Adult, Female	9	73	T2	Ukraine: Crimea	13.05.2007
AO 103 Juv. Male	0	3	PGUppC10	Poland: Romanów	14.04.2007
AO 108 Adult, Female	3	19	P1	Poland: Śrem	10.07.2007
AF 2 Adult, Male	1	14	P7	Poland: Lublin	03.05.2001
AF 3 Adult, Female	5	21	SGUppC10	Poland: Biebrza	17.09.2003
AF 4 Juv. Male	1	11	P8	Poland: Lublin	18.05.2002
AF 7 Adult, Female	4	25	T4	Slovenia: Bohinjska Bela	08.06.1999
AF 46 Juv. Male	1	14	P1, P8	Germany: Osten	21.07.2004
AF 64 Adult, Female	11	176	T2, T4	Iceland: Ostisland	17.06.2002
AF 65 Adult, Male	1	20	S1	Greece: Crete	23.07.2006

Abbreviations: AF – Short-eared Owl; Af – alula feathers; AO – Long-eared Owl; l, r – left, right wings; P – primaries, PGUppC – primary greater upper-wing coverts; S – secondaries; SGUppC – secondary greater upper-wing coverts; T – rectrices.

## Data Availability

The collected mite specimens data used to support the findings of this study are available from the corresponding author upon request. Data sets utilised for this research were retrieved from AMU-DABE. This feather material is currently deposited in the Natural History Collections and Department of Avian Biology and Ecology, Faculty of Biology, Adam Mickiewicz University, Poznań, Poland (UAM). All collected mite specimens are deposited in the Adam Mickiewicz University, Department of Animal Morphology, Poznan, Poland (UAM).
